# A positioning pillow to improve lumbar puncture success rate in paediatric haematology-oncology patients: a randomized controlled trial

**DOI:** 10.1186/1471-2407-9-21

**Published:** 2009-01-15

**Authors:** Perrine Marec-Bérard, Alvine Bissery, Kamila Kebaïli, Matthias Schell, Françoise Aubert, Ségolène Gaillard, Muriel Rabilloud, Behrouz Kassaï, Catherine Cornu

**Affiliations:** 1 Service d'Oncologie Pédiatrique, Institut d'Hémato-Oncologie Pédiatrique, Centre Léon Bérard, Lyon, France; 2 Hospices Civils de Lyon, Service de Biostatistique; CNRS, UMR 5558, Villeurbanne; Université Claude Bernard, Laboratoire Biostatistique Santé, Lyon, F-69003, France; 3 Hospices Civils de Lyon, Service d'hématologie pédiatrique; Lyon, F-69000 France; 4 INSERM, CIC201, EPICIME, Lyon, F-69000 France; CHU Lyon, Service de Pharmacologie Clinique, Lyon, F-69000 France; Univ Lyon, UMR 5558, Lyon, F-69000 France

## Abstract

**Background:**

Lumbar punctures (LPs) are common in children with cancer. Although pain management during the lumbar puncture has been well standardized, dealing with stress and anxiety is not well addressed yet. Our objective was to evaluate the potential improvement of the LP success rate using a positioning pillow, to ensure maximum lumbar flexion, and allow paravertebral muscles to relax, in children who are awake, with either conscious sedation or no sedation.

**Methods:**

Children aged 2–18 years undergoing LP were randomly assigned to a positioning pillow or no intervention. The primary outcome was the rate of success, i.e. achieving the LP (sampling or injection) at the first attempt, without bleeding (RBC < 50/mm^3^). The secondary outcomes included: the child's pain, assessed by a self-administered visual analogical scales (VAS) for children over 6 years of age; the parents' and caregivers' perception of the child's pain; the satisfaction of the children, the parents, the caregivers and the physician. The child's cooperation and the occurrence of post-LP syndrome were also evaluated.

**Results:**

124 children (62 in each group) were included. The LP pillow tended to increase the success rate of LPs (67% vs. 57%, p = 0.23), and decreased the post-LP syndromes (15% vs. 24%, p = 0.17) but the differences were not statistically significant. In children over 6-year of age (n = 72), the rate of success was significantly higher in the pillow group (58.5% vs. 41.5%, p = 0.031), with a tendency to feel less pain (median VAS 25 vs. 15 mm, p = 0.39) and being more satisfied (84.4% vs. 75.0%, p = 0.34).

**Conclusion:**

Overall results do not demonstrate a benefit in using this pillow for lumbar punctures. This study results also suggest a benefit in the sub group of children over 6-year of age; this result needs confirmation.

**Trial Registration:**

The trial was registered with Clinical Trials.gov (number NCT00775112).

## Background

Lumbar punctures (LP) are commonly performed for diagnosis or treatment purposes in children with cancer or haematological diseases. Anaesthesia or deep sedation is not recommended in France for lumbar punctures [1]. Because of lack of anaesthesia, appropriate body posture, muscle relaxation, and quietness of the child are important determinants of the success of the LP. When LPs are performed in a good position, pain is well controlled by local anaesthesia. A good positioning of the child usually requires the presence of at least 3 attendees: one to hold the child, one to perform the LP, and a third person to serve or help the performer

In a paediatric population, LP causes anxiety and stress [2], leading to a high rate of samples being traumatic or hemorrhagic. Repeated attempts to obtain a successful LP will, in turn, aggravate anxiety [3]. Evans et al. have shown that 70% of patients undergoing LP have post traumatic haemorrhage with a red blood cell counts (RBC) ranging from 1–5/mm^3 ^(27%) to more than 50/mm^3 ^(24%) [4]. Needle size, thrombocytopenia, repeated or recent LPs are among factors that affect its quality [3,5,6]. The rate of successful LPs in our hospital within the last 2-year reached 70%. Success was defined as a LP achieving its objective at the first attempt (sampling or injection), and a RBC ≤ 50/mm^3^.

Although awareness in controlling pain during invasive procedures has increased, evidence based pain management remains insufficient.

Non pharmacological, cognitive, and behaviour-based techniques such as hypnosis, comfortable environment and distractions are currently used to control pain [7-10].

To decrease discomfort and apprehension, and to improve the position of the patient during the LP, we developed a positioning pillow. This pillow allows children to remain in an appropriate position throughout the LP procedure, and to be relaxed. The device is currently in use in our centre, and seems useful for the nursing staff and the children. A randomized controlled study was performed to estimate the success rate of LPs using the LP pillow compared to the usual procedure. Secondary outcomes were pain, anxiety, post-LP syndrome defined according to Spencer et al. description [11], and the satisfaction of the children, the parents and the caregivers.

## Methods

### Participants

Children aged 2 to 18 years undergoing an LP were eligible for inclusion. Children who had already participated or used the LP pillow, who had a medical condition (orthopaedic anomaly) contraindicating the use of the LP pillow or whose parents refused consent were excluded.

Participants undergoing LPs were randomly assigned to "LP pillow" or "no intervention". A permuted-block algorithm was used for randomization, and participants were stratified according to allocation centre. Concealed allocation was centralized by a phone call to the coordination centre after eligibility check and baseline data collection.

### Intervention

**Figure 1:**
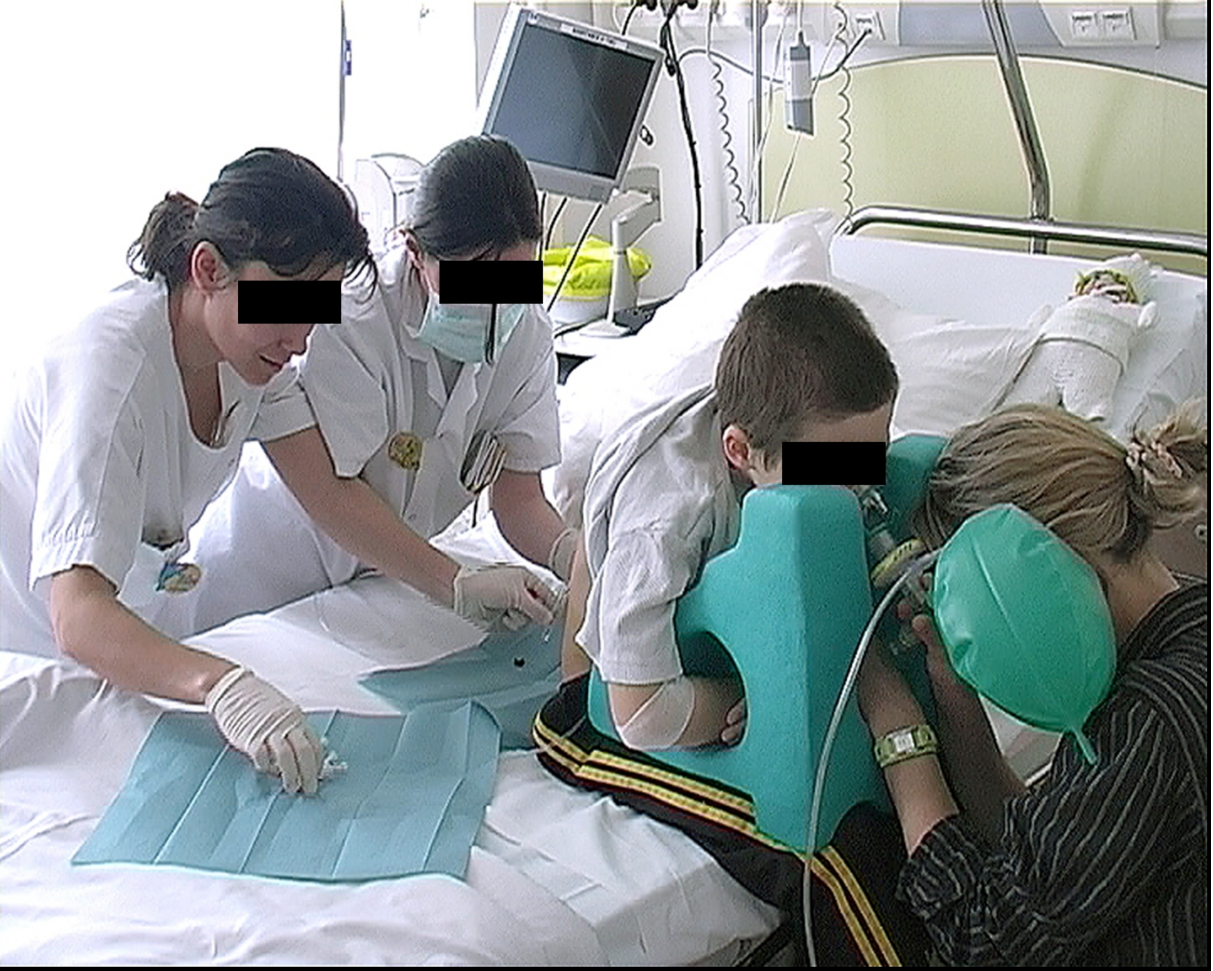
**Illustration: The LP pillow is placed on the thighs of the child; it includes side supports for the head, openings to allow the child to breathe and speak, to touch the child's hands and forearms and be able to administrate nitrous oxide, N2O-O2**. Props supporting the patient's arms maintain the cervical column in a neutral position.

LPs were performed according to the routine clinical practice of participating centres, in a sitting position of the child, using local anaesthesia, and mild sedation, the child being awake in both groups. The LP pillow is made of polyethylene microcellular foam, coated with rubber to facilitate decontamination. It is placed on the thighs of the child who was sitting with his trunk leaning forward. This position ensures a maximum lumbar flexion. The trunk can rest on the pillow allowing paravertebral muscles relaxation. The body axis and the spinal column are perfectly maintained symmetrical in the sagittal plane (Figure 1).

The pillow includes side supports for the head; the face remains uncovered in order to allow the child to breathe, speak comfortably and to facilitate nitrous oxide administration. Aides hold the patient's arms along the cervical column in a neutral position and an opening allows parents to touch their child's hands and forearms. To ensure effective stabilization, splints positioned at the bottom side of the LP pillow immobilize the child's thighs. Four LP pillow sizes were available for the study (for 2–6 year, 6–10 year, 10–15 year and 15–18 year). Sterilisation was performed using surface disinfection after each use.

The study was performed with experienced operators having performed more than 50 LPs before. The minimal required platelet count prior to LP was 50,000/mm^3^.

### Outcomes

The primary outcome was the success or the failure of the LP. LP was rated successful when it achieved its purpose (sampling and/or treatment) at the first attempt, without visible haemorrhage or with RBC < 50/mm^3 ^in the cerebrospinal fluid (CSF) sample (cytological analysis). When one of these criteria was missing, LPs were rated as failure.

Secondary outcomes included: the child's pain, evaluated by self-administered visual analogical scales (VAS) for children over 6 years of age; the parents' and caregivers' perception of the child's pain; the satisfaction of the child, the parents, the caregivers and the physician who performed the LP; the cooperation of the child rated with the "LeBaron Scale", a 8-item scale, rated by an observer to evaluate the child's anxiety based on its behaviour [12]; the incidence, the symptoms and the length of the post-LP syndrome assessed 48 hours after the LP by a phone interview; post-LP syndrome was defined as a bifrontal, occipital, neck, or upper shoulders location headache, with onset during the first 24 or 48 hours, characterised by postural worsening in upright position, coughing, straining and which alleviates while lying down, of mild intensity to prostrating, with possible associated symptoms: photophobia, nausea, loss of appetite, diplopia [11]. To be able to estimate if the pillow seemed convenient or not, we also counted the number of attendees (parents and/or caregivers) present and the length of the LP procedure.

The data collection included the drugs used (anaesthetic, sedative and analgesic drugs); the needle size (19G, 20G, 22G); the general medical state of the child evaluated with Lansky score, a performance status rating scale used in children with cancer [13]; the platelet count; the number of previous LPs; the date of the LP and the satisfaction with the last LP (verbal scale), the practitioner's experience in performing LP, the presence of the parents, the aim of the LP (diagnosis, therapeutic monitoring, treatment injection) and the amount of CSF removed (drops).

The study protocol was approved by the institutional review board of Lyon A – Hôtel-Dieu on the 8^th ^June 2004, and was conducted in accordance with the Helsinki Declaration. All parents gave a written informed consent before their child participated in the study. Children were asked to give written consent when fully able to understand the proposed procedure.

Data management and quality control were performed by CLININFO S.A. (France).

### Sample size and statistical analysis

The protocol initially included a total of 80 children. The sample size calculation was based on a 70% success rate in the control group and 95% in the LP pillow group, with 80%-power and 0.05 two-sided significance level. After inclusion of the 40^ieth ^child and based on the success rate in the control group, the sample size was re-estimated and increased to 124 children (62 per group). In order to achieve this sample size, inclusion period was extended from 12 to 24 months.

Analyses were performed according to the intention to treat principle. All patients were kept in their randomization group, regardless of subsequent protocol deviations. Depending on the nature of the variable, Wilcoxon rank or Fisher's exact tests were used to compare results between groups. A logistic regression model was fitted to the platelet count and the number of LPs prior to entering the study. An exploratory subgroup analysis was performed in children over 6 years of age. For the main outcome, comparisons with p-value <= 0.05 were considered significant. The STATA 9.2 software (SatatCorp 2005. Stata Statistical Software: Release 9.0 College Station, TX: Satat Corporation) was used to perform statistical analyses. Statistical analyses, sample size calculation and generation of the sequence of allocation were performed at the Lyon University Hospital Department of Biostatistics.

## Results

**Figure 2:**
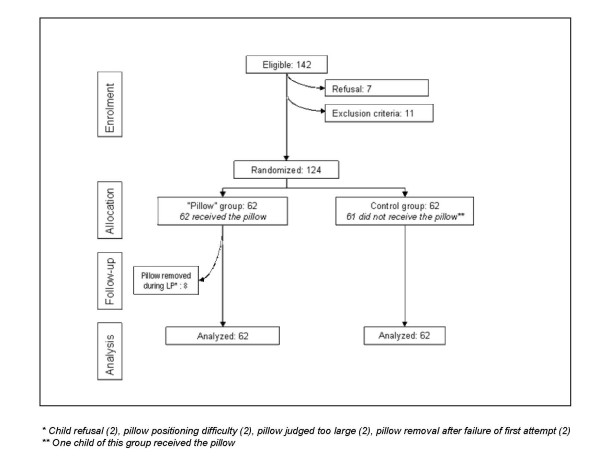
**flow diagram of the progress through the study**.

Between July 2004 and September 2006, 124 children, 62 in the pillow group and control group were included. Twenty four children were included in centre 1 (paediatric oncology service), and 100 children in centre 2 (paediatric haematology), in the paediatric hemato-oncology Institute, Lyon, France. Because of technical problems, cerebrospinal fluid samples could not be analyzed in three patients (two in the control group, one in the pillow group). One participant in the control group was given a pillow (protocol deviation) (Figure 2)

Analysis of the baseline characteristics of participants (table 1) shows no major differences between randomized groups. Concomitant therapies to alleviate pain or stress were identical in both groups: 30% received premedication with Hydroxyzine 1 mg/Kg, 98% received local anaesthesia with a lidocaïn/prilocaïn patch, and 94% received N_2_0-0_2 _therapy.

**Table 1:** Baseline characteristics of the participants

	Pillow group n = 62	Control group n = 62	P
	median (min, max)		
Age (years)	6.63 (2.18–17.48)	7.70 (2.02–16.68)	0.60
Status, Lansky or Karnofsky score (%)	100 (40–100)	100 (40–100)	0.86
Platelet count	189.5 (11–660)	201.5 (24–609)	0.63
Number of previous LP	2 (0–16)	2 (0–16)	0.82
	N (%)		
Male	38 (61.3)	37 (59.7)	0.85
Previous LP	55 (88.7)	57 (91.9)	0.54
***Description of the LP***	(n, %)		
Premedication	21 (33.9)	15 (24.2)	0.24
EMLA^®^	61 (98.4)	61 (98.4)	1
Entonox	57 (91.9)	60 (96.8)	0.24
Purpose of LP			0.3
Diagnosis*	7 (11.3)	7 (11.3)	
Disease follow-up	10 (16.1)	4 (6.5)	
Treatment injection	45 (72.6)	51 (82.3)	
	Median (min-max)		
LP duration (minutes)	4 (1–33)	4 (2–19)	0.52^§^
Number of attempts	1 (1–5)	1 (1–4)	0.98^§^
Amount of CSF** withdrawn (drops)	20 (10–34)	20 (20–40)	0.3^§^
Number of attending persons	4(2–7)	4 (2–5)	0.5^§^
LP performed in sitting position (first attempt), (n, %)	62 (100)	62 (100)	

* Diagnostic LPs can be performed for relapse diagnosis, or to verify the efficacy of treatment. It is therefore possible for children to have a diagnostic LP and several previous LPs

** CSF = cerebrospinal fluid

^§ ^Mann & Whitney test

With LP pillow, the rate of success, the patient's pain, the satisfaction of the child, the parent's and the nursing staff, the LP duration and the number of attending persons were not significantly improved (table 2).

**Table 2:** Outcomes, intention to treat analysis

	Pillow group n = 62	Control group n = 62	P
Primary outcome: successful LP (n, %, n = 121)	41 (67.2)	34 (56.7)	0.23
Only one attempt (n, %)	50 (80.7)	50 (80.7)	-
Objective of LP reached (n, %)	60 (96.8)	60 (96.8)	-
Macroscopic haemorrhage (n, %)	10 (16.1)	5 (8.1)	0.17
Microscopic haemorrhage (RBC > 50/mm^3^, n, %)	42 (67.7)	47 (75.8)	0.49
Operating physician satisfaction: nb very satisfied or satisfied (n, %, n = 124)	49 (79)	52 (83.9)	0.49
Child anxiety (LeBaron scale): median (min.-max, n = 124)	4 (0–33)	3.5 (0–23)	0.28
Child satisfaction: nb "very satisfied" or "satisfied" (n, %, available in children over 6 years, n = 47, 46)	27 (84.4)	27(75)	0.34
Nursing team satisfaction: nb "very satisfied" or "satisfied" (n, %, n = 124)	50 (81)	49 (79)	0.82
Parents satisfaction (n, %, n = 75 answers)	24 (72.7)	34 (81)	0.39

Post-LP syndrome (n, %, n = 124)	9 (14.5)	15 (24.2)	0.17

The overall rate of success was 62%, 67% in the LP pillow and 57% in the control group. Reasons for LP failure included i) need for more than one attempt to achieve the LP (19%), ii) LP goal not reached (3%), and iii) CSF haemorrhage (21%, either microscopic or macroscopic).

Eight (33%) children in centre 1 and 28 (77.8%) in centre 2 received premedication, 24 (100%) in centre 1 and 98 (98%) in centre 2 received local anaesthesia using an EMLA^® ^patch and 19 (79%) in centre 1 and 98 (98%) in centre 2 received nitrous oxide (ENTONOX^®^). Clinical routine practices were different between the two centres regarding the attendance of care givers: 3 persons on average in centre 1, and 4 in centre 2 attended the LPs. One parent was present in 79% of cases in centre 1, and in 57% in centre 2 (p = 0.004). The platelet count was lower in centre 2 compared to centre 1: 305,083/mm^3 ^(SD 166,714), vs 177,085 (SD 106,903), p = 0.0011.

We performed a post-hoc analysis in the subgroup of 72 children aged over 6-year (58%). In this sub-group, LP success rates were significantly higher with the LP pillow (58.5% vs. 41.5%, p = 0.031). This subgroup seem more satisfied (84.4% vs. 75.0%, p = 0.344) with the pillow (Table 3).

**Table 3:** Subgroup analysis, outcomes in children over 6 years of age

	Pillow group N = 38	Control group N = 34	P
Primary outcome: successful LP (n, %)	24 (63)	17 (50)	0.031
Children's pain (Visual analogical scale*, mm, median (min-max)^a^	15 (0–91)	25 (0–90)	0.39
Child satisfaction: nb very satisfied or satisfied (n, %, children over 6 years, n = 36)	27 (84.4)	27(75)	0.34

*: this scale has been validated for children above 6 years only

## Discussion

We performed a randomized controlled trial to estimate the benefit of the LP pillow in improving the success rate of LPs and in reducing patient discomfort. Our hypothesis was that the LP pillow by improving the child's position, would increase the rate of successful LPs, and decrease pain, especially when LPs are performed without deep sedation.

There was no statistically significant difference between LP rate of success with and without pillow. Results were also indeterminate for all secondary criteria. Even though our results are inconclusive, LP Pillow tends to improve consistently the main outcomes. The rate of successful LPs tended to be higher in the pillow group (68% vs. 57%, p = 0.23), with fewer bleeding (19% vs. 23%, p = 0.48), and post-LP syndromes (15% vs. 24%, p = 0.17). Children seemed to declare less pain with the pillow (median VAS 15 vs. 25 mm, p = 0.39), whereas caregivers seemed to perceive more pain (median VAS 17.5 vs. 10 mm, p = 0.16) and more anxiety (median score 4 vs. 3.5, p = 0.28) in children treated with the pillow. Lumbar punctures with local topical anaesthesia and sedation with MEOPA^® ^produce usually fairly low pain scores [1]. This might explain why we were not successful to reduce the pain scores with LP pillows.

In terms of satisfaction, children seemed to be more (84% vs 75%, NS), caregivers equally (81% vs 79%, NS), and operators less satisfied (79% vs 84%, NS) with the pillow.

Nine (7%) patients who were in the pillow group had the pillow removed when difficulties occurred while performing the LPs. These difficulties occurred mostly in very young patients, since eight of them were less than 6 years of age. These patients were all in centre 2. This centre had little experience with the pillow before the study, but recruited most patients. Family attendance does not influence the child's anxiety, pain, satisfaction, nor the nursing staff's satisfaction, nor the LP success rate, as published before [14].

Optimising human resources use by decreasing the number of attendees during LP procedure was a potential advantage of the pillow. We believe that the number of attendees was not modified during the study in any group, because no specific action was taken to encourage physicians to reduce this number.

The rate of success with LP pillows was significantly higher in the subgroup of children over 6-year of age. This was, however, a post hoc analysis, and the study was not stratified on children's age. This sub-group analysis was planned after care givers reported that the pillow appear not to be very useful for young children. The cut-off age was fixed at 6 years since it was the cut-off for validated pain and satisfaction scales. We think that the shape, size or smoothness of the device might be insufficiently adapted to smaller children. For younger children, the device seems too large and too hard, and technical improvements are necessary. Moreover, LPs might be easier in younger children without supporting devices.

We are not aware of any other published studies evaluating medical devices used to improve LPs through positioning management.

We based our number of necessary subject calculation on the LP rate of success reported in previous studies [4-6,14,15]. At our hospital the rate of traumatic LPs (20% within the last two-year) was consistent with those reported in the literature. We expected a 70% success rate in our centre which is very similar to the rate of success described in the literature when LPs are performed without deep sedation/anaesthesia. But only 60% of LPs succeeded in the control group. With this rate of success, and an 11% difference between groups, more than 300 patients per group would have been required to provide an 80% statistical power to the study. One explanation might be that most children were recruited in centre 2, and had a lower platelet number (305 (SD 166) vs 177 (SD106), p = 0.0011).

Because of the nature of the intervention, it was not possible to warrant blind intervention. Therefore, the assessment of some secondary endpoints, e. g. the child's anxiety on the Le Baron scale, level of pain and satisfaction evaluated on VASs were potentially biased.

Conducting clinical trials to evaluate interventions to alleviate pain in children with cancer is difficult, because of the very limited numbers of patients. Recruiting 124 patients was therefore quite difficult and demonstrating a significant difference was very challenging. The pillows are still in use in the services who performed the study.

## Conclusion

Overall results do not demonstrate a benefit in using this pillow for lumbar punctures. This study results also suggest a benefit in the sub group of children over 6-year of age. Should further studies be performed, they should target this age range.

## Competing interests

The *Centre Léon Bérard *holds the patent of the Pillow.

The authors declare that they have no competing interests.

## Authors' contributions

PMB: conceived of the study, participated in its design and coordination, recruited patients in centre 1, and helped to draft the manuscript. AB: performed the statistical analysis. KK: recruited patients in centre 2. MS: recruited patients in centre 1, and validated endpoints

FA: attended LPs, and called the families for 48h-follow-ups in centre 2, for data collection SG: participated in study coordination. MR: supervised the statistical analysis. BK: participated in study coordination, drafted and reviewed the manuscript. CC: conceived the study, participated in its design and coordination, and drafted the manuscript. All authors read and approved the final manuscript,

## Pre-publication history

The pre-publication history for this paper can be accessed here:

http://www.biomedcentral.com/1471-2407/9/21/prepub

## References

[B1] Schmitt C Theobald S Fabre N Kasparian C Seblain C Boutard P Mansuy L Marec-Berard P Monvillers C Munzer M Orbach D Sakiroglu O Tumerelle G Annequin D Carbajal R Luu M Ricard C Torloting G Wood C [Standards, options and recommendations for the management of procedure-related pain (lumbar puncture, bone marrow aspiration or biopsy, blood sampling) in children patients with cancer (summary report)] Bull Cancer 2006 93 8 805 811 16941757

[B2] Bouffet E Douard MC Annequin D Castaing MC Pichard-Leandri E [Pain in lumbar puncture. Results of a 2-year discussion at the French Society of Pediatric Oncology] Arch Pediatr 1996 3 1 22 27 10.1016/S0929-693X(96)80004-2 8745822

[B3] Chordas C Post-dural puncture headache and other complications after lumbar puncture J Pediatr Oncol Nurs 2001 18 6 244 259 10.1053/jpon.2001.28454 11719905

[B4] Evans RW Complications of lumbar puncture Neurol Clin 1998 16 1 83 105 10.1016/S0733-8619(05)70368-6 9421542

[B5] Breuer AC Tyler HR Marzewski DJ Rosenthal DS Radicular vessels are the most probable source of needle-induced blood in lumbar puncture: significance for the thrombocytopenic cancer patient Cancer 1982 49 10 2168 2172 10.1002/1097-0142(19820515)49:10<2168::AID-CNCR2820491031>3.0.CO;2-O 7074532

[B6] Howard SC Gajjar AJ Cheng C Kritchevsky SB Somes GW Harrison PL Ribeiro RC Rivera GK Rubnitz JE Sandlund JT de Armendi AJ Razzouk BI Pui CH Risk factors for traumatic and bloody lumbar puncture in children with acute lymphoblastic leukemia JAMA 2002 288 16 2001 2007 10.1001/jama.288.16.2001 12387652

[B7] Ellis JA Spanos NP Cognitive-behavioral interventions for children's distress during bone marrow aspirations and lumbar punctures: a critical review J Pain Symptom Manage 1994 9 2 96 108 10.1016/0885-3924(94)90162-7 8021541

[B8] Hageman-Wenselaar LH [Hypnosis for pain control during lumbar puncture and bone marrow aspirations in children with cancer] Tijdschr Kindergeneeskd 1988 56 3 120 123 3413764

[B9] Polkki T Vehvilainen-Julkunen K Pietila AM Nonpharmacological methods in relieving children's postoperative pain: a survey on hospital nurses in Finland J Adv Nurs 2001 34 4 483 492 10.1046/j.1365-2648.2001.01777.x 11380715

[B10] Rape RN Bush JP Psychological preparation for pediatric oncology patients undergoing painful procedures: a methodological critique of the research Child Health Care 1994 23 1 51 67 10.1207/s15326888chc2301_4 10132664

[B11] Spencer HC Postdural puncture headache: what matters in technique Reg Anesth Pain Med 1998 23 4 374 379 discussion 384-377. 10.1016/S1098-7339(98)90009-8 9690589

[B12] LeBaron S Zeltzer L Assessment of acute pain and anxiety in children and adolescents by self-reports, observer reports, and a behavior checklist J Consult Clin Psychol 1984 52 5 729 738 10.1037/0022-006X.52.5.729 6501658

[B13] Lansky SB List MA Lansky LL Ritter-Sterr C Miller DR The measurement of performance in childhood cancer patients Cancer 1987 60 7 1651 1656 10.1002/1097-0142(19871001)60:7<1651::AID-CNCR2820600738>3.0.CO;2-J 3621134

[B14] Nigrovic LE McQueen AA Neuman MI Lumbar puncture success rate is not influenced by family-member presence Pediatrics 2007 120 4 e777 782 10.1542/peds.2006-3442 17908735

[B15] Nigrovic LE Kuppermann N Neuman MI Risk factors for traumatic or unsuccessful lumbar punctures in children Ann Emerg Med 2007 49 6 762 771 10.1016/j.annemergmed.2006.10.018 17321005

